# Comparison of IgG diffusion and extracellular matrix composition in rhabdomyosarcomas grown in mice versus *in vitro* as spheroids reveals the role of host stromal cells

**DOI:** 10.1038/sj.bjc.6600270

**Published:** 2002-05-03

**Authors:** C de L Davies, D A Berk, A Pluen, R K Jain

**Affiliations:** Department of Radiation Oncology, Massachusetts General Hospital and Harvard Medical School, Boston, Massachusetts, MA 02110, USA

**Keywords:** diffusion, extracellular matrix, tumours, spheroids, stromal cells

## Abstract

The tumour extracellular matrix acts as a barrier to the delivery of therapeutic agents. To test the hypothesis that extracellular matrix composition governs the penetration rate of macromolecules in tumour tissue, we measured the diffusion coefficient of nonspecific IgG in three rhabdomyosarcoma subclones growing as multicellular spheroids *in vitro* or as subcutaneous tumours in dorsal windows *in vivo*. In subcutaneous tumours, the diffusion coefficient decreased with increasing content of collagen and sulphated glycosaminoglycans. When grown as multicellular spheroids, no differences in either extracellular matrix composition or diffusion coefficient were found. Comparison of *in vitro* *vs* *in vivo* results suggests an over-riding role of host stromal cells in extracellular matrix production subjected to modulation by tumour cells. Penetration of therapeutic macromolecules through tumour extracellular matrix might thus be largely determined by the host organ. Hence, caution must be exercised in extrapolating drug penetrability from spheroids and multilayer cellular sandwiches consisting of only tumour cells to tumours *in vivo*.

*British Journal of Cancer* (2002) **86**, 1639–1644. DOI: 10.1038/sj/bjc/6600270 www.bjcancer.com

© 2002 Cancer Research UK

## 

The extracellular matrix (ECM) is a potent barrier to the delivery and penetration of complex biopharmaceuticals such as monoclonal antibodies, therapeutic proteins, or genes. Due to the elevated interstitial fluid pressure in tumours (Boucher and Jain, 1992), the therapeutic agents must reach the tumour cells by interstitial diffusion. It is not clear whether the network of fibrillar collage or the glycosaminoglycan (GAG) gel plays the most important role in limiting the diffusion of macromolecules through the ECM, and what the role of the host stromal cells is. Netti *et al* (2000) found that the amount of collagen in tumours correlated inversely with the diffusion coefficient of macromolecules, and collagenase treatment of the tumour increased the diffusion coefficient. Pluen *et al* (2001) provided further support for the role of collagen by measuring diffusion in tumours grown in subcutaneous tissue and the cranium. Hyaluronidase, on the other hand, is reported to reduce the diffusion coefficient of albumin in lung interstitium (Qiu *et al*, 1999).

We postulated that diffusion of macromolecules is hindered both by the collagen network and by the hydrophilic gel of GAG and proteoglycans. To test this hypothesis we used three distinct clones with different degrees of differentiation and ECM (Groeschel *et al*, 1994) derived from a rat rhabdomyosarcoma, growing as multicellular spheroids *in vitro* or subcutaneously in dorsal chambers in mice *in vivo*. The production of ECM in tumours is the result of an active interaction between the tumour cells and stromal cells of the host (Gullino and Grantham, 1962; Knudson *et al*, 1984), whereas the extracellular matrix of multicellular spheroids is solely produced by the tumour cells. Comparison of the ECM composition in tumours growing as xenografts *in vivo* and as multicellular spheroids *in vitro*, is likely to reveal the influence of the host stromal cells on ECM composition and transport properties.

## MATERIALS AND METHODS

### Spheroids

Three clonal subpopulations A, B, and C derived from the same dimethyl-benzanthracene-induced rhabdomyosarcoma in rat (Gerharz *et al*, 1988) were kindly provided by Dr H Gabbert (Department of Pathology, University of Dusseldorf, Germany). The cell lines were grown at 37°C and 5% CO_2_ Dulbecco's Modified Eagle's Medium with 4500 mg glucose ml^−1^, supplemented with 10% heat-inactivated foetal calf serum and 100 U ml^−1^ penicillin-streptomycin (all from Sigma).

Spheroids were initiated using the liquid overlay technique. 2·10^5^ cells were grown for 5 days in tissue culture flasks (75 cm^2^) coated with 1% agarose. Approximately 20 spheroids with a diameter less than 200 μm were placed inside 50 mm long glass capillary tubes (Vitro Dynamics Inc, Rockaway, NJ, USA) with sides of 0.2 mm×4 mm, and cultivated for an additional day to allow the spheroids to adhere to the surface. The medium was removed by capillary forces, replaced with medium containing 0.3 mg ml^−1^ fluorescein-IgG, and incubated for 4–6 h.

### Tumours

Tumour cells were implanted either subcutaneously in dorsal skinfold chambers (Leunig *et al*, 1992) or injected subcutaneously in the flank of SCID mice. The transparent window chamber allowed non-invasive diffusion measurements. When the tumours growing in the dorsal chambers filled the chambers, 200 μl of 2 mg ml^−1^ fluorescein-IgG was administered by tail vein injection. Clone A grew somewhat faster than clone B and C, filling the chamber with a tumour diameter of approximately 10 mm 6–9 days after implantation, whereas 9–12 days were needed for clones B and C. The tumours growing subcutaneously in the flank were used for measuring ECM constituents, as the amount of tumour tissue from dorsal chambers was not sufficient for such measurements. Sections of paraffin-embedded tumours and multicellular spheroids were stained with haematoxylin-eosin. All animal experiments have been carried out with ethical committee approval. The ethical guidelines that were followed meet the standards required by the UKCCCR guidelines (Workman *et al*, 1998).

### Diffusion measurements

Interstitial diffusion was measured by fluorescence recovery after photobleaching (FRAP), 24 h after i.v. injection of fluorescein labelled IgG, as described by Berk *et al* (1993; 1997). Goat γ-globulin (Sigma, St. Louis, MO, USA) was labelled with the fluorophore fluorescein-EX using a protein labelling kit from Molecular Probes (Eugene, OR, USA).

An argon laser (model 2020; Spectra-Physics, Mountain View, CA, USA) at 488 nm, was focused onto the tissue through the microscope objective (×20, NA 0.4) to form a circular spot with nominal diameter of 40 μm. After a brief exposure to laser illumination, wide-field epifluorescence images were projected onto an intensified CCD camera (model 2400; Hamamatsu Photonics, Hamamatsu City, Japan), digitised, and stored at a rate of five images s^−1^ for 130 s. Photobleaching recoveries were quantified by spatial Fourier analysis (Berk *et al*, 1993, 1997). The diffusion coefficient was given as the ratio relative to diffusion in solution to emphasise the reduction of the diffusion coefficient in tissue. The use of ratio also effectively eliminates the potential systematic error associated with the measurement technique.

### Disintegration of tissue

The tumour tissue had to be solubilised in order to measure ECM constituents. Spheroids and tumours cut in pieces were placed in a digest buffer (50 mg tissue ml^−1^). The buffer consisted of 125 μg ml^−1^ papain (Sigma) in 0.1 M Na-phosphate (Merck, Darmstadt, Germany), 5 mM Na_2_EDTA (Merck, Darmstadt, Germany), and 5 mM cysteine-HCl (Sigma), pH 6.0. Tumour tissue and spheroids were finely dispersed with a homogenizer (Polytron; Brinkmann Instrument, Westbury, NY, USA), and incubated in the digest buffer for 18 h at 60°C.

### GAG content

The total amount of GAG was determined by the reaction of uronic acid with carbazole as described by Bitter and Muir (1962). Briefly, 0.5 ml solubilised tissue diluted in water saturated with benzoic acid was carefully layered onto 3 ml of sulphuric acid at 0°C, shaken, and boiled for 10 min. After cooling to room temperature, 100 μl carbazole (Sigma) reagent was added, the samples shaken, boiled for 15 min and cooled to room temperature. The absorbance was measured using a spectrophotometer (model UV-1201; Shimadzu, Kyoto, Japan) at 530 nm, and the concentration of GAG equivalent to uronic acid was determined from the standard curve.

Sulphated glycosaminoglycans (s-GAG) was measured using the Blyscan proteoglycan and s-GAG assay (Biocolor Ltd, Belfast, Ireland), which is based on the specific binding of the cationic dye, 1,9-dimethylmethylene blue (Farndale *et al*, 1986). Briefly, 10 μl of diluted solubilised sample and 1 ml Blyscan dye reagent was mixed for 30 min at room temperature and centrifuged for 10 min. The polysaccharide-dye complex precipitated and was resolved in 1 ml dissociation reagent. The absorbance of bound dye was measured at 656 nm. The concentration of s-GAG equivalent to uronic acid was obtained using the standards of the Blyscan assay with the assay for total GAG.

Concentration of hyaluronan (HA) was estimated as the difference between total GAG and s-GAG, both expressed equivalent to uronic acid.

### Collagen content

The collagen content was determined by measuring hydroxyproline according to Woessner (1961). Briefly, 300 μl of solubilised sample was hydrolysed by adding HCl to a final concentration of 6N. The samples were hydrolysed at 110°C for 18 h, followed by neutralisation using 2.5 N NaOH. Hydroxyproline was oxidised by adding chloroamine T, mixed and left at room temperature for 20 min. Cloramine T (Sigma) was destroyed by adding 1 ml 3.15 M percloric acid (Merck, Darmstadt, Germany), mixed and left at room temperature for 5 min. Finally, 1 ml p-dimethylaminobenzaldehyde (Sigma) was added, mixed and placed at 60°C for 20 min. The absorbance was measured spectrophotometrically at 557 nm. The concentration of hydroxyproline was determined from the standard curve, and concentration of collagen estimated by assuming 6.94 μg collagen μg^−1^ hydroxyproline (Jackson and Cleary, 1976).

### Statistical analysis

Statistical comparisons of data were performed using the one-way analysis of variance, ANOVA. The significance criterion of *P*<0.05 was used. Correlation between parameters was calculated as the Person product moment correlation coefficient. Regression analysis was done by least squares fit. All statistical analysis was performed using Minitab (Minitab Inc., State Collage, PA, USA).

## RESULTS

### ECM composition

**Figure 1:**
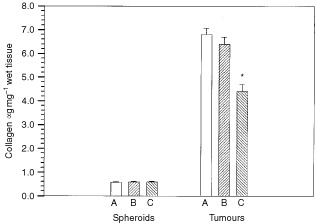
Collagen content in clone A, B, and C of BAHAN rhabdomyosarcoma growing as tumours or as multicellular spheroids. Each value represents the mean and standard error of 8–12 measurements of spheroids and 10 tumours. Significant difference between the clones is indicated (*).

The three rhabdomyosarcoma clones exhibited different ECM composition when grown *in vivo*. In contrast, no difference in any of the measured ECM constituents was observed when the cells grew as multicellular spheroids (Figures 1

and 2

**Figure 2:**
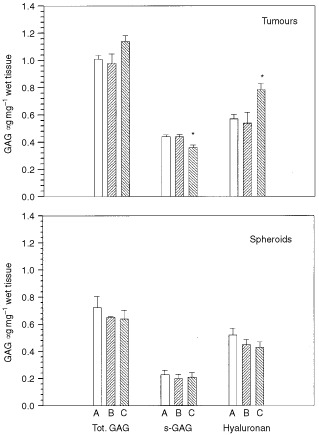
GAG content in clone A, B, and C of BAHAN rhabdomyosarcoma growing as tumours (upper panel) or as multicellular spheroids (lower panel). GAG, s-GAG and hyaluronan are all expressed as equivalent mass to uronic acid. Each value represents the mean and standard error of four measurements of spheroids and 12–14 tumours. Significant difference between the clones is indicated (*).

). *In vivo*, clone C had approximately 30% less collagen (Figure 1), and about 50% more HA than clone A and B (Figure 2). No difference was found in the level of total GAG. Thus, the content of s-GAG, the difference between total GAG and HA, was lower in clone C compared to clone A and B (Figure 2). Plotting collagen *vs* s-GAG showed a positive correlation, and the correlation coefficient was estimated to be 0.98 (*P*=0.10).

The ECM composition measured in tumours growing subcutaneously in the flank was confirmed by immunofluorescence in tumours growing in dorsal chambers. Sections of tumours were stained using a primary antibody against collagen type I and the immunofluorescence quantified by confocal laser scanning microscopy (data not shown).

**Figure 3:**
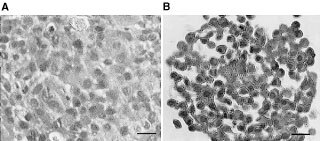
Haematoxylin-eosin stained sections of paraffin-embedded tumours (**A**) and multicellular spheroids (**B**). Images were obtained using 60× oil immersion objective. Bar=30 μm.

Haematoxylin-eosin stained sections of paraffin embedded tumours and spheroids showed a higher cell density in tumours growing in dorsal chambers compared to spheroids (Figure 3

). In spheroids the cell density was rather heterogeneous with large areas without cells, whereas the tumours showed a more homogeneous cell density distribution. This was observed for all three clones.

### Interstitial diffusion coefficients

**Figure 4:**
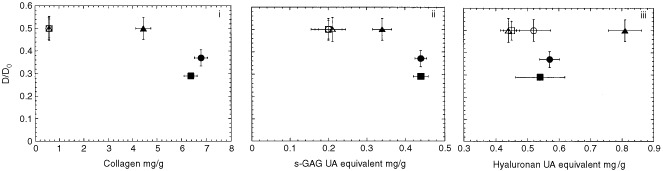
Diffusion coefficient D of IgG in BAHAN rhabdomyosarcoma growing as tumours (closed symbols) or as multicellular spheroids (open symbols) relative to D_0_ in solution. Clones A, B, and C are represented by (•/○), (▪/□), and (▴/▵), respectively. D/D_0_ is plotted as a function of collagen (i), s-GAG (ii), and hyaluronan (iii). s-GAG and hyaluronan are expressed as equivalent mass to uronic acid. Each value of D represents the mean and standard error of 4–7 tumours or spheroids with 5–12 FRAP measurements per tumour or spheroid.

Therapeutic macromolecules must diffuse through the ECM, consisting of a fibrillar protein network embedded in a hydrophilic gel of GAG, in order to reach the tumour cells. The diffusion coefficient of IgG was measured by FRAP. The ratio between the diffusion coefficient in tumours growing in dorsal windows and the diffusion coefficient in free solution varied from 0.30 to 0.50 (Figure 4

). *In vivo*, IgG diffusion in clone C tumours was about 30 and 70% higher compared to clone A and B tumours, respectively. For multicellular spheroids, no significant differences in the diffusion coefficient of IgG were found between the three clones, and the ratio of diffusivities was reduced to about 50% (Figure 4).

### No difference in matrix content or diffusion between the subclones grown as spheroids

Although the tumour cells generated an extracellular matrix with measurable collagen and GAG content when grown *in vitro* as multicellular spheroids, no difference among the three clones grown as spheroids was found with respect to collagen content or GAG content (total GAG, HA, s-GAG). Correspondingly, there was no difference in diffusion coefficient among the three clones. In all subclones the diffusion coefficient of IgG was the same as in clone C growing as xenograft *in vivo*.

### Correlation between matrix content and diffusion in tumour xenografts

In contrast to the spheroids, the tumour xenografts did exhibit significant differences in ECM composition and transport properties among the clones. The diffusion coefficient measured *in vivo* decreased with increasing concentration of collagen (*r*^2^=0.72; *P*=0.35), and s-GAG (*r*^2^=0.86; *P*=0.24), and increased with increasing concentration of HA (*r*^2^=0.92; *P*=0.18). Clone C having the lowest amount of collagen and s-GAG, and the highest amount of HA was found to have the highest diffusion coefficient (Figure 4). These data therefore collectively suggest that IgG diffusion is principally hindered by the collagen network and/or the s-GAG.

## DISCUSSION

In seeking to establish a quantitative relationship between tumour ECM properties and the penetration of high-molecular weight therapeutics, we examined the interstitial diffusion coefficient of IgG as a function of collagen, GAG, s-GAG, and HA content. An important novel aspect was to study the impact of host stromal cells by comparing ECM content and diffusion in tumours growing in mice *in vivo* or as spheroids *in vitro*.

### Correlation between matrix content and diffusion

An inverse, but weak, correlation between collagen content and diffusion coefficient was found and is consistent with findings in various human and murine tumours (Netti *et al*, 2000), as well as in matrices *in vitro* (Shenoy and Rosenblatt, 1995). Increasing the concentration of collagen supposedly decreases the distance between neighbouring fibrils, thus decreasing the pore size of the matrix, thereby increasing the frictional interactions between the IgG and the matrix. In accordance with this, greater diffusional hindrance was recently demonstrated in tumours with higher levels of collagen type I organised into fibrils (Pluen *et al*, 2001). In the *in vivo* study by Netti *et al* (2000), the interpretation was complicated by the substantial differences in cell morphology, collagen organisation, and the lack of knowledge about the origin of the matrix material from tumour versus stromal cells. In the present study using three clones isolated from the same rhabdomyosarcoma, it seems reasonable to assume that the collagen was produced by host cells in response to interactions with the growing tumour cells, and that there is less differences in matrix assembly and organisation between these three tumour clones compared with tumours with different origin. Our results are therefore a significant confirmation of the correlation between collagen content and diffusional hindrance.

Previous comparison of different human tumour xenografts did not reveal a correlation between the diffusion coefficient and s-GAG content (Netti *et al*, 2000). We found an inverse, but weak, correlation with s-GAG. In solutions of HA or sulphated proteoglycans, however, the diffusivity of macromolecules is reported to decrease exponentially with increasing concentration of HA (Laurent *et al*, 1963) or sulphated proteoglycans (Preston and Snowden, 1973). This relationship has also been explained theoretically based on the stochastic model of diffusion (Ogston and Sherman, 1961). Thus, our results indicate that HA and s-GAG have different effects on diffusion in complex *in vivo* systems compared to in pure solution, or the discrepancy is due to the large differences in HA and proteoglycan concentrations in the solutions compared to tissue. The inverse correlation between diffusion and s-GAG might be explained by reduced sulphation of GAG as the s-GAG content decreases. Sulphation increases the charge density, and increases the affinity of GAG for collagen, fibronectin and laminin (Iozzo, 1985). The reduced affinity between GAG and the protein network of collagen and fibronectin might change the assembly and structure of the ECM thereby increasing the pore size of the ECM. The requirement for GAG sulphation to obtain collagen binding has also been demonstrated by Comper and Laurent (1978) who found that collagen binds to chondroitin sulphate, dermatan sulphate, heparan sulphate and heparin, but not to HA or keratin. Consistent with this, we found that the content of collagen and s-GAG correlated, which might reflect that the s-GAG content is determined by the amount of collagen available to bind it. This supports the idea of Netti *et al* (2000) that the proteoglycans require a stabilising solid matrix to fully hinder the transport through ECM, and that collagen has this stabilising and binding capacity. Another explanation is that a reduction in the overall negative charge density also reduces the repulsive electrostatic forces between the GAG's and the negatively charged IgG, thereby increasing both the available volume and the diffusivity (Maroudas, 1970). Further studies of diffusion in tumours with the same collagen content, but with different s-GAG compositions are needed to discriminate the role of s-GAG from that of collagen.

The tumour cells growing as spheroids generated an ECM with collagen and GAG content about 10 and two times lower than in xenografts *in vivo*, respectively. Although the distribution of collagen is different between the three clones growing as spheroids (Groeschel *et al*, 1994), we found no differences in the amount of collagen or GAG (total GAG, s-GAG, HA). Correspondingly, no difference in diffusion coefficient among the three clones was seen. The low level of ECM constituents in spheroids, suggests a higher diffusion coefficient than in tumours. However, the diffusion coefficient of IgG in spheroids was the same as found for clone C growing in the dorsal chamber. There are two possible explanations for this apparent paradox: (1) Below a certain threshold level of ECM constituents, the diffusion coefficient does not increase further, i.e. even a low content of ECM constituents hinders molecular transport considerably, or (2) the extracellular space and structure are radically different in tumours compared to spheroids. The spheroids had larger areas of extracellular space and lower cell density than the tumours. Thus, the low diffusion coefficient in spheroids was not due to compact spheroids. It is the structure of the interstitium rather than a high cell density that causes the hindrance. The steric exclusion and tortuosity of pathways through the interstitium are thus greater in the multicellular spheroids than in the tumours. The diffusion is not solely dependent on the content of ECM constituents, but the structure of these constituents also plays a crucial role. The lack of a vascular network in spheroids might also contribute to the difference in ECM structure.

### Indirect influence of stromal cells on the transport parameters

The ECM constituents in tumours are normally produced by the stromal cells of the host, whereas the tumour cells may modulate the synthesis rate to ensure a favourable environment for the malignant cells (Iozzo, 1988). The tumour cells affect the synthesis rate either directly or indirectly by releasing cytokines. However, it has been reported that tumour cells, and especially sarcoma cells might have the capacity to synthesise the ECM constituents themselves. Tumour cells growing as three-dimensional multicellular spheroids possess this capacity. Multicellular spheroids being an *in vitro* system of intermediate complexity between monolayer cultures and tumours may contain an ECM at an intermediate level (Davies *et al*, 1997). Consistent with this, we also found less collagen and GAG in the spheroids compared to the tumours.

The difference in ECM content of tumours observed when growing as xenografts *in vivo* was not maintained when the tumours cells were grown *in vitro*. This indicates that the stromal cells themselves or the interaction between the stromal cells and the tumour cells play an important role in inducing the difference in ECM composition and as a consequence influence molecular transport. This is consistent with the slower diffusion of various macromolecules found in tumours growing in dorsal chambers compared with cranial windows. The slower diffusion in dorsal chambers was associated with a higher density of stromal cells and higher level of collagen type I organised into fibrils (Pluen *et al*, 2001). The use of multicellular spheroids or multilayer cellular sandwiches incorporating normal fibroblasts might thus be a more accurate tumour model than spheroids containing only tumour cells. The importance of the tissue structure and microenvironment has also been reported by Bissell *et al* (1999) who reported that the structure of the tissue is dominant over the genome due to the bidirectional pathways that connect the cellular microenvironment and the genome.

### Clinical implications

In the design of macromolecule-based therapies, ranging from protein-based to gene therapy, the macromolecules must be able to penetrate through tumour tissue. It would appear overly simplistic to assume a single value for penetration resistance in all tumours. The present work, taken together with other recent studies (Netti *et al*, 2000; Pluen *et al*, 2001), begins to provide a rationale for assessing the relative penetrability of different tumours depending on the tumour type and the site of growth. This work again implicates the collage network and collagen-binding GAGs as factors in hindering the interstitial diffusion of macromolecules. Multicellular spheroids and multilayer cellular sandwiches/membranes are being used to test the penetrability and effectiveness of therapeutics (Fracasso and Colombatti, 2000; Padron *et al*, 2000; Johnson *et al*, 2001). Since these *in vitro* systems do not have the host stromal cells, the present work shows that caution must be exercised in extrapolating the results obtained from these systems to tumours *in vivo*.
